# Suicidal Ideation After Discharge From Psychiatric Hospital: Momentary Assessment Study

**DOI:** 10.2196/88745

**Published:** 2026-07-31

**Authors:** Wenche Ryberg, Lien My Diep, Mathilde Husky, Roar Fosse

**Affiliations:** 1 Department of Mental Health Research and Development, Division of Mental Health and Addiction, Vestre Viken Hospital Trust, Pb 800, Drammen, Buskerud, 3004, Norway, 47 03525; 2 Research Support Services, Oslo Centre for Biostatistics and Epidemiology (OCBE), Oslo University Hospital, Oslo, Norway; 3 Bordeaux Population Health Research Center, Active Team, INSERM U1219, University of Bordeaux, 3 ter, place de la Victoire, 33076, Bordeaux, France

**Keywords:** suicidal ideation, suicidal behavior, suicide attempt, ecological momentary assessment, longitudinal assessment, postdischarge period, psychiatric hospital

## Abstract

**Background:**

High suicide risk is observed after discharge among patients hospitalized with suicidal thoughts and behaviors. Suicidal ideation (SI) is a well-known precursor to suicide attempts (SAs) and suicide, and it is a central indicator of subjective distress.

**Objective:**

This study aimed to examine patient characteristics and predischarge symptoms that might (1) distinguish between participants with and without postdischarge SI, (2) predict peak SI frequency, and (3) predict the intensity and longitudinal trajectory of SI.

**Methods:**

Before discharge, patients hospitalized due to high suicide risk were screened for diagnosis (MINI 7.0.2 [Mini International Neuropsychiatric Interview] and SCID-5-PD [Structured Clinical Interview for *DSM-5* Personality Disorders]), general symptom severity (Outcome Questionnaire-45 [OQ-45]), depression (Patient Health Questionnaire-9 [PHQ-9]), SI (Suicide Status Form-IV [SSF-IV] and Beck Scale for Suicide Ideation), and suicidal behaviors (Suicide Attempt Self-Injury Count). Twenty male and 16 female adult participants installed a mobile app on their smartphones and reported their level of SI on a 5-point Likert scale (1=not at all to 5=very much) 5 times per day for 10 days after discharge. Associations between baseline characteristics and postdischarge SI were analyzed using logistic regression, median regression, and mixed-effects linear regression.

**Results:**

SI was present in 49.2% (528/1073) of all completed surveys. Women reported SI more frequently than men (*χ*^2^_1_=63.39; *P*=.001). No baseline variables differentiated participants who later did (28/36, 77.8%) and did not (8/36, 22.2%) report momentary SI after discharge. Participants hospitalized due to a high risk of suicide (12/36, 33.3%), compared with those hospitalized following an SA (24/36, 66.7%), reported SI more often (*χ*^2^_1_=22.64; *P*=.001) and had a higher frequency of peak SI scores (β=17.74, 95% CI 8.56‐26.91; *P*=.001). Moreover, the variation in the temporal trajectory of postdischarge SI was characterized by large differences between participants (intraclass correlation coefficient [ICC]=0.682), while the intensity of SI did not change over the 10-day period at the group level (β=0.03, 95% CI –0.01 to 0.06; *P*=.09). Predischarge scores on the SSF-IV chronic subscale, including hopelessness, self-hate, and psychological pain, predicted the intensity of SI after discharge (β=0.13, 95% CI 0.05-0.20; *P*=.002). No baseline variables predicted the trajectory of SI.

**Conclusions:**

SI after discharge was common, varied substantially between participants, and may be more prevalent than previously reported, particularly in groups with high morbidity and prior SAs. The SSF-IV may be a valuable tool for treatment planning and assessment during the inpatient stay.

## Introduction

The risk of suicide is exceptionally high during the first days and weeks after an inpatient stay in mental health services [1] and is estimated to be 200 to 300 times greater than that of the general population. Among the high-risk inpatient population, patients with a history of suicide attempts (SAs) or suicidal thoughts and behaviors represent the group at greatest risk of suicide death after discharge [2-5]. Suicidal ideation (SI) is a well-known precursor of SA and suicide [6,7] and is the third most common risk factor for suicide after psychiatric hospitalization and SA [8].

To reduce suicide rates following discharge from inpatient wards, a central task is to understand the factors that influence suicide risk during that time. However, limited knowledge exists on how patients experience the postdischarge period. The transition from hospital to home may itself be a major challenge that leaves patients at continued risk of suicide [9]. Qualitative research has shed light on how this transition may be experienced by patients. In a systematic review, Mutschler et al [10] reported that successful transitions were promoted by themes such as safety, supported autonomy, and engagement in reintegration activities. In contrast, poverty, interpersonal difficulties, and social stigma were barriers that hampered safe transitions. Moreover, Redding et al [11] described unpreparedness or disagreement about the decision to discharge as potential drivers of increased suicidal thoughts and behaviors. Lived-experience research has shown that patients may experience both feelings of unsafeness and unpreparedness, as well as mixed emotions of joy and anxiety after discharge, and that suicide attempters may experience difficulties with social adjustments [12]. A renegotiation with life may be challenging, as feelings of shame and embarrassment, together with ongoing depression and SI, may lead to isolation and social withdrawal [12].

A crucial next step is to identify the subset of patients who experience continued difficulties after hospital discharge. Most current knowledge on risk factors for SI, suicidal behaviors, and suicide after discharge has been gathered from large registry, epidemiological studies, and retrospective accounts sampled through interviews or questionnaires [1,2,4,5,13]. One method that is particularly suited to capturing the inner landscape of lived experience and SI is ecological momentary assessment (EMA) [14]. By applying intensive sampling procedures, EMA makes it possible to capture the phenomenology of SI, as well as its proximal risk factors. Across psychological theories, the shifting and dynamic nature of SI has been conceptualized and acknowledged as a complex phenomenon shaped by situational factors, cognitive appraisals, affective intensity, and interpersonal relations. In recent years, a growing body of empirical research using EMA methods has shown that SI fluctuates substantially within individuals and across short periods of time [15-18]. A few studies have investigated phenotypes of momentary SI during hospitalization to unfold risk profiles and predictors of suicidal thoughts and behaviors. Results, however, remain inconsistent. Kleiman et al [19] reported that a profile characterized by high mean intensity and low variability of momentary SI was strongly associated with a recent SA, reflecting high risk and severity. Similarly, Wang et al [20] found that acute changes and shifts in momentary SI during hospitalization were associated with an increased risk of SA after discharge. Homan et al [21] identified what they interpreted as a high-risk profile, where high mean levels and high variability of momentary SI were associated with higher baseline levels of hopelessness and trauma exposure.

A small set of prior EMA studies has also reported on the frequency, duration, intensity, and variability of SI following discharge, with varying findings [22-32]. These studies have started to investigate fine-grained associations between characteristics of momentary SI and patients’ concomitant, as well as ultra-short-term predictive, momentary cognitions, feelings, behaviors, relationships, and social contexts during the postdischarge period. However, proximal predictors of momentary SI over the medium-term period, from before to after discharge, remain largely unexplored. To our knowledge, no previous studies have examined whether predischarge symptoms and patient characteristics can distinguish between individuals who do and do not experience momentary SI after discharge, despite evidence suggesting a generally low likelihood of experiencing postdischarge momentary SI following inpatient care [23]. Moreover, prior research has not investigated whether predischarge symptom severity and patient characteristics predict subgroups characterized by recurrent high-intensity momentary SI, beyond what is captured by high mean levels or overall variability. Likewise, little is known about how predischarge symptoms influence the subsequent temporal intensity and trajectories of momentary SI after discharge. In this context, examining how predischarge characteristics shape subsequent postdischarge momentary SI may illuminate our understanding of how SI later unfolds and inform clinical decisions, potentially strengthening suicide prevention measures.

We report findings from a Norwegian study of momentary SI that followed participants for 10 days after discharge from psychiatric inpatient care. We aim to (1) characterize key features of momentary SI, (2) examine the temporal progression of momentary SI, and (3) explore how baseline characteristics and variables are associated with momentary SI after discharge. Regarding the latter, we specifically consider (1) differences in baseline variables between participants with and without momentary SI, (2) the relationship between baseline variables and the frequency of peak scores on momentary SI postdischarge, and (3) the association between baseline variables and the intensity and trajectory of postdischarge momentary SI.

## Methods

### Study Design

In this naturalistic observational study of momentary SI, we recruited patients from publicly funded inpatient acute wards in specialized mental health care services in a health region in Norway from August 2022 to February 2024. Acute psychiatric hospitalization in Norway is initiated in situations where a patient with a severe mental health condition or severe mental health crisis may pose a danger to themselves or others or is unable to function. Hence, individuals admitted to advanced acute specialist care within the mental health system represent those with the most acute need for treatment and protection.

### Participants

Adult patients (aged 18‐65 years) admitted to psychiatric acute wards following an SA or due to severe risk of suicide (participants hospitalized due to imminent risk of suicide) were invited to participate. In addition, the project included only patients who were planned for outpatient treatment within specialized care (see below, the security protocol). Exclusion criteria were inability to provide informed consent, presence of psychosis, neurodevelopmental disorders (including autism spectrum disorder), intellectual disability (or dementia), and inability to speak Norwegian.

A power analysis was conducted assuming an intraclass correlation coefficient (ICC) of 0.50. With 50 participants and 5 daily assessments over a period of 10 days, the study was adequately powered to detect significant associations with SI, with medium to large effects in the multivariable mixed-effects model. The power analysis was conducted using Power Curves for Multi-Level Studies (shinyapps.io). The research team met with 58 patients for the initial interview. Among them, 8 did not meet the inclusion criteria, 8 declined participation, and 6 withdrew their consent prior to their discharge because they felt that the protocol was too demanding. Altogether, 36 participants were included.

### Procedures

Hospital staff informed eligible participants about the study. The first author (WR) administered all screening interviews, except for 2 that were conducted by a research assistant. The first author also administered all diagnostic assessments. The EMA app was developed and programmed in cooperation with the University of Oslo’s IT Department, Section for App Development. The EMA protocol was implemented on smartphones, with information collected 5 times each day over a 10-day period following discharge. In addition, participants were interviewed at 3 time points: before discharge, 3 weeks after discharge, and 3 months after discharge. The EMA survey consisted of 26 to 28 questions that took 2 to 4 minutes to complete. See Multimedia Appendix 1 for a presentation of the application’s workflow and interface.

The EMA app was programmed to start sending surveys on the morning after participants left the ward. Participants were provided with training on using the app and were instructed to contact their therapist or an emergency center in case of a crisis, as the application was not an online help service. EMA assessments were distributed randomly within 5 equal-sized time intervals between 10 AM and 10 PM. Participants received a push notification from the app when a survey was ready to be filled out. Surveys were available for a 30-minute time window, and prompts were not repeated if they were not answered. A supportive message with advice and contact information for helplines and emergency centers was displayed if the participant indicated a high intensity of SI. Information shared in the application was not shared with therapists. Although there is little evidence that intense assessments of SI are harmful even in high-risk populations [17,33], the project followed a security protocol during the EMA assessment period to ensure participant safety [34]. One measure was that all participants compiled a safety plan before discharge. Another principle was that all participants had access to follow-up services provided by the hospital’s outpatient services after discharge to ensure patient welfare. Participants’ therapists were informed about the project and their patient’s participation. The research team contacted participants within 48 hours after discharge to provide consultation on the EMA app and answer any questions. A researcher response team was accessible to participants during the EMA assessment period for guidance in using the app when needed. Staff contact information was provided to participants in the mobile application (Multimedia Appendix 1). Surveys completed on the mobile application the previous day were monitored each morning. If participants had not submitted any surveys, the team contacted them to check on their well-being and determine whether support was needed. Moreover, if participants had a response style indicating, for example, very high scores on questions mapping SI and low control thereof, or if a shift in responses indicative of increased symptom severity was observed, the team would contact the participant to check with them and provide support as needed. Support consisted of advising the participant to contact their therapist, agreeing to have the researcher inform the therapist or other support services, or deciding to end study participation if necessary. Furthermore, if a participant was hospitalized during the assessment period, the research team (in collaboration with the participant and their therapist at the inpatient ward) assessed whether the mobile application was perceived as a burden or felt harmful in any way and decided whether the assessment could continue or not.

### Measures

#### Descriptive Characteristics

We gathered general information about each participant from interviews and the electronic patient registry. At baseline, we collected information on gender, education, marital status, living situation, children, work status, previous hospitalizations in mental health care, and the number of days in the current hospitalization. At 3 months follow-up, we counted the number of outpatient sessions the participants had received and the number of rehospitalizations since discharge.

#### Mental Disorders

Before discharge, participants were screened using the Mini International Neuropsychiatric Interview (version 7.0.2) [35]. If this was not feasible, the interview was conducted at the earliest possible time point within the first few days following discharge. We administered the Structured Clinical Interview for *DSM-5* Personality Disorders (SCID-5-PD) [36] after 3 weeks or 3 months, depending on participants’ symptom severity and status, to ensure the validity of the screening results.

#### Symptom Severity, Depression, and Suicidal Cognition

Beck Scale for Suicide Ideation*-*Current (BSSI-C) inquires about the worst level of SI the interviewee has experienced during the last 2 weeks. It consists of 19 items rated on a 0 to 2-point Likert scale, with a total score of 38. Higher total scores on the BSSI-C indicate higher suicide risk. The BSSI-C is structured as an interview and is considered to be a reliable measure of SI [37].

Suicide Status Form-IV (SSF-IV) is a multipurpose tracking and evaluation form for suicide-related cognitions. It inquires about the respondents’ current experiences of psychological pain, hopelessness, stress, agitation, and self-hate. Items are rated on a 5-point Likert scale (from low to high), with a total score of 25. The SSF-IV can be divided into 2 subscales or factors: acute and long-term or *chronic* SI. Items in the latter subscale contain 3 components: “psychological pain,” “hopelessness,” and “self-hate,” with a total score of 15, whereas the first subscale is represented by the 2 items “stress” and “agitation,” with a total score of 10 [38-40]. In addition, the SSF-IV asks the respondents to self-assess their suicide risk on a scale from 1 to 5, ranging from “low risk” to “very high risk” of suicide. The SSF-IV was administered both before discharge and after 3 weeks and 3 months. The form has been reported to have well-established psychometric properties in terms of reliability and validity [38].

We mapped participants’ previous experiences with self-inflicted harmful behaviors (including SAs) using the Suicide Attempt Self-Injury Count (SASIC) [41]. SASIC is a shorter version of the more extensive Suicide Attempt Self-Injury Interview (Linehan MM and Comtois K, Lifetime parasuicide history, unpublished work, University of Washington, 1996), which includes both a lifetime version and a recent version. We administered the lifetime version at baseline and the recent version at 3 weeks and 3 months.

We used the Outcome Questionnaire-45 (OQ-45) to measure general symptom severity before discharge and after 3 months. The OQ-45 consists of 45 questions about subjective discomfort, interpersonal relations, and social role performance. Items are rated on a 0 to 4-point Likert scale ranging from “never” to “always,” with a total possible score of 180. The questionnaire is sensitive to change, has demonstrated sound psychometric properties, and has been validated in a Norwegian sample [42,43].

The participants completed the Patient Health Questionnaire-9 (PHQ-9) [44] before discharge. This is a very brief assessment consisting of 9 questions about the severity of depression, with each question scored on a 4-point Likert scale ranging from 0 to 3, representing “not at all” to “almost every day,” with a total score of 27. The PHQ-9 is considered a reliable measure [45]. A score above 20 usually indicates severe depression when diagnostic criteria are fulfilled.

The *EMA protocol* consisted of 26 to 28 items inquiring about current thoughts and emotions, context or relations, and whereabouts, which were rated on Likert scales or with radio buttons. In this paper, we focused our analyses on the EMA item assessing current SI: “*I am thinking about taking my life*” [46], which was scored on a 5-point-scale, ranging from 1 (“not at all”) to 5 (“very much”) and distributed to participants 5 times a day over the first 10 days after discharge. Scores above “1” were defined as a positive report of SI. Single items are commonly used in the EMA literature to measure subjective state phenomena and may be particularly sensitive to change over short periods of time [17,47,48]. Assessments with single items reduce participants’ burden compared to administering a full traditional multi-item scale (eg, time spent) and may improve compliance and response rates. As the EMA item assessing SI was a single item, internal consistency (eg, Cronbach α) cannot be estimated. However, in a previous investigation, items from a larger EMA battery were psychometrically investigated for convergent validity and reliability [46], including the single item “*I think about taking my life.*” Reports indicated substantial within-person variability and acceptable prompt-level reliability [46].

### Analyses

#### Research Question 1: What Are the General Characteristics of Momentary SI?

Momentary SI and baseline variables were first characterized using descriptive statistics, including means with SDs, medians with quartiles, and frequencies with percentages, along with chi-square and Fisher exact tests.

#### Research Question 2: What Is the Temporal Trajectory of Momentary SI?

**Figure 1.**
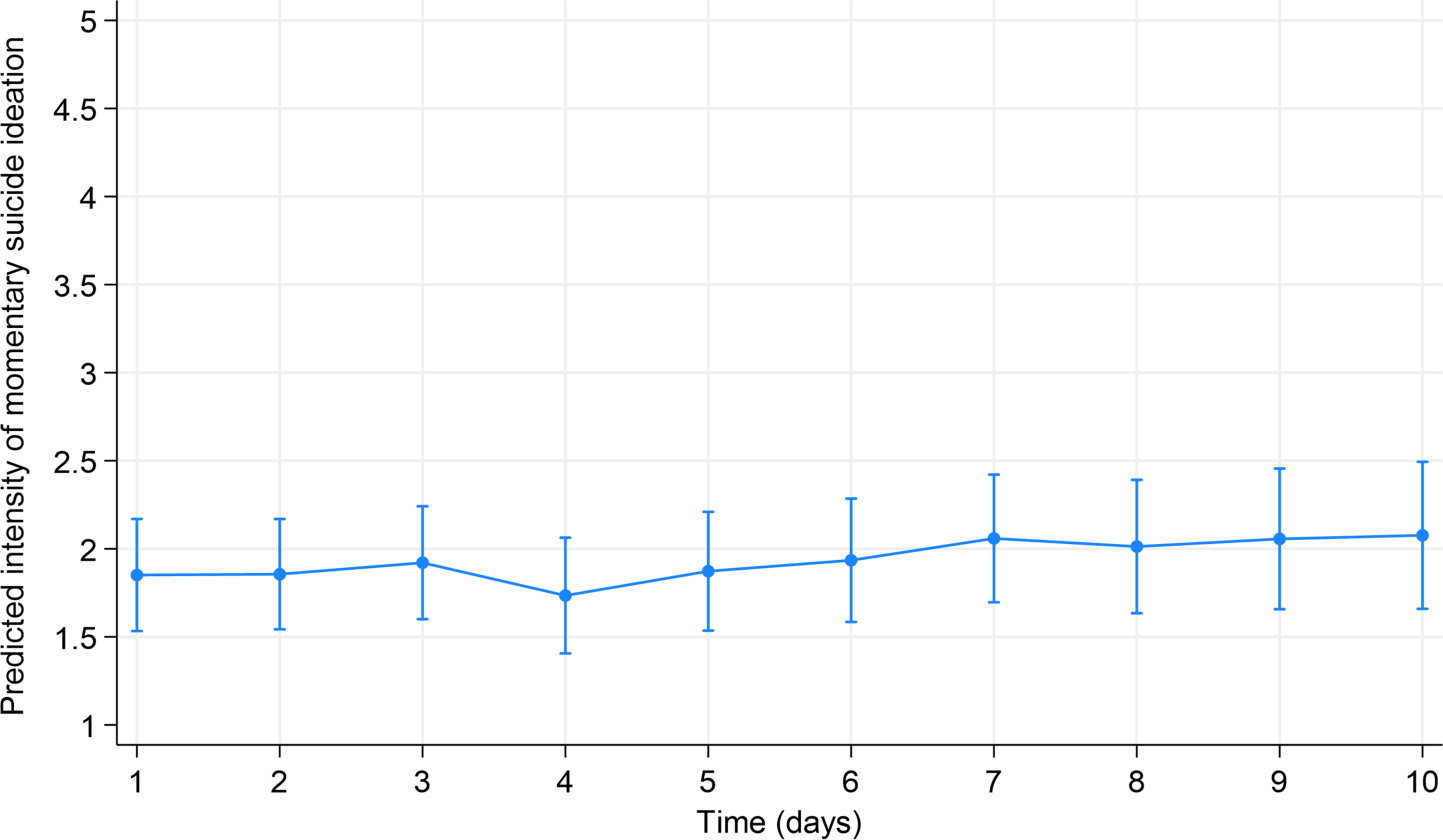
Temporal pattern of momentary suicidal ideation across days. In this figure, we used a mixed-effect linear model where time (days) was defined as a categorical variable to estimate marginal predicted means with 95% CIs for each day (see *Methods* section).

We examined the temporal trajectory both in terms of time point of the day and over days in separate models. First, we examined how much of the overall variance in momentary SI was divided between and within participants by estimating ICC by running an intercept-only model. Next, a linear mixed-effects model for continuous outcomes was used to fix approximately 5 data points per participant over 10 days. In the analyses of momentary SI trajectory over days, we conducted 2 linear mixed-effects models: one random intercept model and one with random intercept and slope model, assuming missing data at random. The random intercept and slope variables were participant identifiers and time, respectively, and an unstructured variance-covariance structure was used for the random intercept and slope model. For the random intercept model, the participant identifier was specified as the random intercept. For the fixed part in both models, the time (days) variable was treated as a continuous variable, assuming SI followed a linear pattern. We compared the 2 linear mixed-effect models using a likelihood ratio test, and the random intercept and slope model was shown to fit our data best. In a separate analysis, we also modeled time (days) as a categorical variable to estimate the average SI score for each day and to explore the day-to-day pattern of change, as illustrated in Figure 1. In sensitivity analyses, we examined whether the temporal development of SI departed from a linear pattern by modeling SI as categorical, continuous, and with a spline model. In the latter, we defined 4-time intervals (3 knots) based on the small curves in Figure 1. The spline model trajectories overlapped substantially with trajectories modeling SI as categorical and linear, indicating that a parsimonious linear model was adequate for further analyses (Multimedia Appendix 2). For the analysis exploring temporal trajectory within days, we repeated the models’ specifications (random intercept and slope) and replaced the continuous variable (days) with the time point of the day.

#### Research Question 3: How Are Baseline Variables Associated With Momentary SI, the Frequency of Peak Values of Momentary SI, and the Intensity and Trajectory of Momentary SI?

In this section, we describe the methods used for each regression model.

Regression model a: We examined baseline differences before discharge between participants who experienced momentary SI during the 10-day period and those who did not. We used logistic regression to explore the association between baseline variables and momentary SI as a binary dependent variable (ie, yes or no momentary SI). The odds ratios (ORs) and 95% CIs were reported for the association.

Regression model b: We examined whether participants’ frequency of peak scores on momentary SI could be associated with baseline characteristics. We first transformed the SI scoring scale from 1‐5 to 0‐4 and continued by extracting the highest SI score for each participant over 10 days. To examine the burden of having high SI scores frequently, we calculated the frequency of the highest SI score during the 10-day period and multiplied that frequency by the highest score for each participant. Thereafter, we explored the association between the weighted frequencies and baseline characteristics using median regression due to the skewed distribution of the frequency data. We provided the estimated ICCs to reflect potential differences in SI variability between participants with an SA and those hospitalized because of suicide risk. Peak scores reflect the most severe symptom state. Although simplified, this approach complements mixed-effects models by emphasizing extreme intensity, a method that has been used in other clinical contexts as an indicator of severity [49,50].

Regression model c: We explored how baseline variables were associated with the intensity and trajectory of momentary SI over the 10-day assessment period. In a series of single mixed-effects linear regression models, each baseline variable, time (days), and baseline variable × time (days) interaction were added and tested. We specified both a random intercept and a random slope and retained the unstructured covariance structure. In all models using mixed-effects linear regression, the restricted maximum likelihood method was used. Given the small sample size, we used a parsimonious approach. Regression coefficients were reported, and *P* values with 95% CIs were provided for additional information.

For all regression models (a, b, and c), we examined each baseline variable separately to evaluate associations with SI while adjusting for gender and age. In the tables, results from unadjusted analyses are provided for additional information. We report only on associations that remained significant after correcting for multiple tests using a Bonferroni procedure.

We examined how missingness on the EMA surveys was associated with baseline characteristics to evaluate whether some participants were more prone to miss prompts than others. We observed that missingness was associated with gender (men missed more surveys compared to women) but not with age, symptom burden, suicide ideation or cognition, or depression. Additional analyses controlling for gender showed no impact. Consequently, we assumed missing at random (MAR) but not missing completely at random (MCAR). Mixed-effects models have been shown to be robust under MAR assumptions. Analyses were performed in SPSS (version 30; IBM Corp) and Stata/SE (version 19.5).

### Ethical Considerations

The study was approved by the Regional Committees for Medical and Health Research Ethics, Region South-East, Norway (2022/393161), and the local Data Protection Officer (22‐04861). Participation was entirely voluntary and contingent on written informed consent. At the end of the study, participants received a NOK 500 (US $50) gift card as a token of appreciation for their contribution.

## Results

### Participant Characteristics

The study sample comprised 36 participants (20 men and 16 women). Among them, 24 (66.7%) participants were hospitalized after an SA (14 men and 10 women). A substantial majority of participants (n=29, 80.6%) reported a lifetime history of SA. Approximately half of the participants (n=18, 50%) had a life partner, lived with someone else, and/or had children. Overall, participants reported moderate to high levels of general symptom severity (OQ-45) and depressive symptoms (PHQ-9; Table 1). Depression was the most common diagnosis and was observed in 50% (n=18) of the sample. Additionally, 17 of 30 (56.7%) participants met the criteria for a personality disorder (PD).

**Table 1.** Baseline characteristics (N=30-36).

Variables	Value
Gender (N=36), n (%)	
Men	20 (55.6)
Age (y), mean (SD); range (N=36)	38.9 (12.4); 18‐59
Social situation (N=36), n (%)	
Lived alone	18 (50)
Had children	19 (52.8)
In a relationship	19 (52.8)
Previous hospitalizations (N=36), mean (SD); range	7.9 (16.8); 0‐68
Suicide attempt (SA^a^) lifetime (N=36), n (%)	29 (80.6)
Participants hospitalized after an SA (N=36), n (%)	24 (66.7)
Days of hospitalization (N=36), mean (SD); median (IQR); range	17.3 (13.5); 12.5 (17); 1-56
Main ICD-10^b^ diagnoses (N=36), n (%)	
Depression	18 (50)
PTSD^c^	7 (19.4)
Adjustment disorder	7 (19.4)
Other diagnosis	4 (11.1)
Personality disorder (PD; N=30), n (%)	
All categories	17 (56.7)
Borderline PD	8 (26.7)
Avoidant PD	5 (16.7)
Other PD^d^	4 (13.3)
Severe habitual self-harm^e^ (N=36), n (%)	8 (22.2)
Suicide ideation (BSSI-C^f^; N=36), mean (SD)	24.7 (4.7)
Symptom severity (OQ-45^g^; N=36), mean (SD)	101.9 (23.3)
Depressive symptoms (PHQ-9^h^; N=36), mean (SD)	20.2 (5.7)
Suicidal cognitions (SSF-IV^i^; N=35), mean (SD)	16.3 (5.1)
SSF-IV acute subscale (N=36), mean (SD)	6.4 (2.2)
SSF-IV chronic subscale (N=35), mean (SD)	9.9 (3.7)
SSF-IV self-assessed suicide risk (N=34), mean (SD)	2.2 (1.3)

a SA: suicide attempt.

b *ICD-10*: *International Statistical Classification of Diseases*, Tenth Revision

c PTSD: posttraumatic stress disorder.

d Other PD categories include antisocial PD and mixed PD (*ICD-10*).

e Subpopulation who were unable to quantify self-harm episodes (<100).

f BSSI-C: Beck Scale for Suicide Ideation-Current.

g OQ-45: Outcome Questionnaire-45.

h PHQ-9: Patient Health Questionnaire-9.

i SSF-IV: Suicide Status Form-IV.

Participants responded to 1073 of 1800 possible EMA surveys, corresponding to a compliance rate of 59.6%. On average, participants delivered 29.8 (SD 12.0; range 7‐49) EMA surveys. Altogether, women delivered 586 (54.6%) of the 1073 surveys. We observed no significant difference in baseline variables between participants with (24/36, 66.7%) and without (12/36, 33.3%) an SA at admission. Table 1 presents a summary of the baseline characteristics.

### Characteristics of EMA-Assessed SI: Presence and Proportions

The presence of SI, defined as a score greater than 1 (“not at all”) on the EMA question “I think about taking my life,” was observed in 528 of 1073 (49.2%) valid surveys. Eight of 36 (22.2%) participants did not report SI at any point during the 10-day intensive assessment period. The remaining 28 out of 36 (77.8%) participants reported momentary SI on at least 1 EMA survey. Women reported the presence of SI more often (325/528, 61.6% surveys) than men (203/545, 37.3% surveys; *χ*²_1_=63.39; *P*=.001). Participants hospitalized without an SA reported more frequent momentary SI (234/399, 58.6% surveys) than those who were hospitalized following an SA (294/674, 43.6% surveys; *χ*²_1_=22.64; *P*=.001).

### Temporal Patterns of Momentary SI

Overall, the variability in momentary SI was characterized by large differences (68%) between participants (ICC=0.682). At the group level, the intensity of momentary SI remained relatively stable over the 10-day assessment period, with no significant increase over time (β=0.029, 95% CI −0.01 to 0.06; *P*=.09). Of importance, we observed considerable variability between participants (random intercept variance σ^2^=0.80, corresponding to SD 0.90). Figure 1 illustrates the temporal pattern of momentary SI.

We observed no statistically significant differences in momentary SI across the 5 daily time points of measurements (β=0.02, 95% CI −0.02 to 0.05; *P*=.28). This indicates that, at the group level, momentary SI was reported with the same intensity during the day.

### Baseline Variables Associated With Momentary SI

#### Differences in Baseline Variables Between Participants With and Without Momentary SI

**Figure 2.**
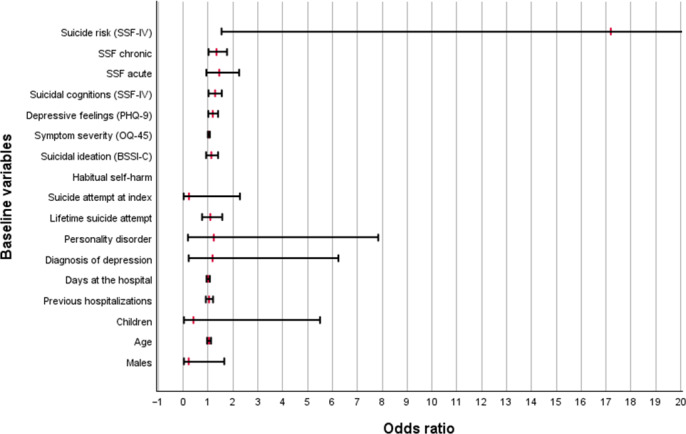
The likelihood of experiencing suicidal ideation (SI) after discharge as a function of scores on baseline variables. The plot visualizes the association between the “suicidal ideation group”—participants who did (n=28) and who did not (n=8) experience momentary SI at any point during the 10-day assessment period—and baseline variables (analyses adjusted for age and gender). The whiskers represent the upper and lower 95% CIs, while the indicators represent the odds ratio (OR). The latter is characterized by a very large 95% CI (OR 17.19, 95% CI 1.54-191.58) and has been partially excluded from the figure for better interpretability. BSSI-C: Beck Scale for Suicide Ideation-Current; OQ-45: Outcome Questionnaire-45; PHQ: Patient Health Questionnaire; SSF-IV: Suicide Status Form-IV.

We observed no significant differences in any baseline symptoms between participants with (28/36, 77.8%) and without (8/36, 22.2%) momentary SI after discharge, after correcting for multiple comparisons (Table 2; Figure 2).

**Table 2.** Participants with and without postdischarge momentary suicidal ideation (SI) and associations.

Variables	With SI (n=28)	Without SI (n=8)	Unadjusted		Adjusted for gender and age	
			OR^a^ (95% CI)	*P* value^b^	OR (95% CI)	*P* value^b^
Men, n (%)	14 (50)	6 (75)	0.33 (0.06-1.94)	.22	0.22 (0.03-1.65)	.14
Age (y), mean (SD)	39.3 (12.1)	37.6 (14.2)	0.99 (0.92-1.05)	.74	1.04 (0.96-1.12)	.34
Children, yes, n (%)	14 (50)	3 (37.5)	0.60 (0.12-3.00)	.53	0.41 (0.03-5.24)	.49
Previous hospitalizations, mean (SD)	8.6 (18.8)	1.9 (1.8)	1.05 (0.92-1.20)	.43	1.04 (0.91-1.19)	.55
Days of hospitalization, mean (SD)	17.8 (15.4)	15.8 (11.0)	1.01 (0.95-1.07)	.73	1.00 (0.94-1.07)	.91
Diagnoses of depression, n (%)	14 (50)	4 (50)	1.00 (0.21-4.81)	>.99	1.18 (0.22-6.24)	.85
Personality disorder, n (%)	13 (46.4)	4 (50)	0.98 (0.18-5.39)	.98	1.23 (0.19-7.84)	.82
Suicide attempt (SA) lifetime, mean (SD)	6 (18.7)	1.9 (1.8)	1.11 (0.77-1.61)	.57	1.09 (0.76-1.57)	.63
SA prior to hospitalization, yes, n (%)	17 (60.7)	7 (87.5)	0.22 (0.02-2.05)	.18	0.23 (0.02-2.28)	.21
Severe habitual self-harm^c^, yes, n (%)	8 (29)	0 (0)	N/A^d^	N/A	N/A	N/A
Suicide ideation (BSSI-C)^e^, mean (SD)	25.3 (4.3)	22.8 (5.9)	1.13 (0.94-1.36)	.19	1.13 (0.92-1.40)	.24
Symptom severity (OQ-45)^f^, mean (SD)	105.0 (24.0)	91.3 (17.8)	1.03 (0.99-1.07)	.15	1.03 (0.99-1.07)	.17
Depression (PHQ-9)^g^, mean (SD)	21.5 (4.8)	15.5 (6.4)	1.21 (1.03-1.41)	.02	1.19 (1.01-1.40)	.04
Suicidal cognitions (SSF-IV)^h^, mean (SD)	17.5 (4.6)	12.0 (4.6)	1.28 (1.05-1.57)	.02	1.28 (1.02-1.55)	.03
SSF-IV acute subscale, mean (SD)	6.9 (2.2)	5.00 (1.5)	1.57 (1.01-2.41)	.04	1.45 (0.93-2.25)	.10
SSF-IV chronic subscale, mean (SD)	10.7 (3.2)	7.00 (4.0)	1.36 (1.05-1.76)	.02	1.34 (1.02-1.76)	.03
SSF-IV self-assessed suicide risk, mean (SD)	2.69 (1.2)	1.13 (0.4)	17.62 (1.85-168.05)	.01	17.19 (1.54-191.58)	.02

a OR: odds ratio.

b Bonferroni correction for multiple tests: 0.05/17=0.00294.

c Subpopulation who were unable to quantify self-harm episodes (<100).

d Not applicable.

e BSSI-C: Beck Scale for Suicide Ideation-Current.

f OQ-45: Outcome Questionnaire-45.

g PHQ-9: Patient Health Questionnaire-9.

h SSF-IV: Suicide Status Form-IV.

#### What Characterizes Participants With Frequent High Peak Scores on Momentary SI?

We observed a significant association between the frequency of peak scores of momentary SI and the reason for hospitalization (SA or suicidality; β=17.74, 95% CI 8.56‐26.91; *P*=.001), with higher mean scores in those hospitalized for suicidality (mean 26.6, SD 48.2) compared with those hospitalized for SA (mean 8.4, SD 12.6). Moreover, baseline levels of self-assessed suicide risk were significantly associated with the peak frequency of SI after discharge (β=6.95, 95% CI 3.95‐9.95; *P*=.001). See Multimedia Appendix 3 for a detailed overview of test statistics. ICC estimate for momentary SI was 0.59 in the SA group, indicating that 41% of the variance in SI over time was observed within participants. At the same time, the ICC estimate for participants hospitalized with suicidality was 0.79, suggesting that a larger proportion of the variance in momentary SI was due to between-participant differences (79%) in this group. Results indicate that momentary SI among the SA group was characterized by a state-dependent pattern, while momentary SI among participants hospitalized with suicidality was characterized by a more trait-like pattern. As sample sizes in the 2 groups were uneven and small, these estimates should be interpreted with caution.

#### Baseline Variables’ Predictive Value for the Intensity and Trajectory of Momentary SI

We conducted a series of separate mixed-effects regression analyses to examine whether baseline variables were associated with the intensity and temporal trajectory of momentary SI over the 10-day assessment period. The SSF-IV chronic subscale was observed to significantly predict the intensity of momentary SI (β=0.13, 95% CI 0.05-0.20; *P*=.002). See Multimedia Appendix 4 for a detailed overview.

## Discussion

### Principal Findings

During the 10-day EMA period after hospital discharge, 28 of 36 (77.8%) participants reported the presence of momentary SI at one or more measurement points, with women reporting experiencing momentary SI more often than men. Participants admitted with and without an SA were equally likely to experience momentary SI at least once, but those hospitalized without an attempt reported it more frequently. The trajectory of momentary SI remained stable over time at the group level, both in terms of days and measurement time points within days, with large variations in the intensity of momentary SI between participants.

We could not identify baseline parameters that differentiated between those who later did and did not experience postdischarge momentary SI. Participants with a high frequency of their own peak score on momentary SI were more likely to have been admitted to the hospital without an SA. Moreover, a higher peak frequency of momentary SI was associated with higher levels of subjectively assessed SI before discharge. The general intensity of momentary SI over the 10-day assessment period was significantly associated with the SSF-IV chronic subscale at baseline, which included hopelessness, self-hate, and psychological pain. No baseline variables predicted the trajectory of momentary SI.

### Comparison With Prior Work

#### Most Participants Experienced Momentary SI

Previous research has indicated that one-third to one-half of patients with a high risk of suicide experience momentary SI after discharge from inpatient psychiatric care [22-29]. The higher prevalence of momentary SI in the present study (28/36, 77.8%) compared to reports from similar settings may reflect a relatively high level of clinical severity in this study. Moreover, overall, few studies have previously been conducted, study samples have been relatively moderate in size, women participants have typically been overrepresented, few suicide attempters have been included, and in some studies, adherence to the EMA protocol has been low [22,23,26-30]. Together, these factors may reflect uncertainty regarding the prevalence of SI after discharge in this population. Our observation of a high prevalence of momentary SI after discharge resonates with findings from a recent epidemiological cross-sectional study. Among 1004 ready-to-be-discharged patients from a psychiatric hospital, 48.9% reported that they experienced active SI [13], indicating that SI is common during the transition from inpatient to outpatient care.

#### Temporal Development of Momentary SI

Several previous EMA studies have reported a relative stability in momentary SI over time at the group level, but with considerable individual variations, in line with what we observed. Armey et al [23] followed suicide attempters, suicide ideators, and other patients (controls) hospitalized in psychiatric care with a 21-day EMA study where they inquired about SI. They observed an overall low likelihood of participants to experience SI, but this likelihood increased over the observation period around days 12 to 15. Wallace et al [29] followed newly discharged patients in a 65-day EMA protocol inquiring about the intensity of SI. They reported that the intensity of SI varied substantially between participants over the assessment period, indicating that momentary SI did not follow a uniform or consistent pattern over time. Sels et al [27] followed a similar patient group in a 4-week protocol and concluded that descriptive plots revealed considerable variability between participants and no clear pattern at the group level in terms of the intensity of momentary SI over time. These findings align with ours and indicate that individual differences may better explain the development of momentary SI than time (days and time within days). Further elaborating on this issue, in a longitudinal EMA study of momentary SI among participants with depression, Oquendo et al [51] found that individuals with highly variable and stress-induced momentary SI (state-like SI) differed from those with stable, persistent momentary SI (trait-like SI). When extrapolating upon their findings, the authors argued that patients with stress-sensitive SI are in the target group for rapid-response and emotion regulation interventions (eg, security planning interventions and dialectical behavioral therapy), while patients with trait-like SI may be in the target group for interventions focusing on maladaptive cognitive patterns. These observations and reflections are relevant in our context, as we observed different variances in SI in participants hospitalized with risk of suicide and in those hospitalized following an SA, respectively (see below).

### Baseline Predictors of Momentary SI

#### Should We Be Less Concerned About Patients Who Deny Momentary SI?

A small subgroup reported no momentary SI after discharge. One interpretation is that these participants quickly resolved their problems upon admittance to the hospital and experienced a drop in symptoms. Such “turning points” have been reported in qualitative studies, explaining how contexts and events may help people change focus [52,53]. On the other hand, participants with no reports of momentary SI were hospitalized for the same duration as other patients, suggesting that clinicians considered them to have a similar need for support. It is possible that this subgroup was less inclined to report, accept, or recognize their own feelings, or that they were characterized by a response style involving denial of problems. Denying SI among high-risk individuals is more common than previously anticipated [54,55]. Multiple pathways to suicidal behavior outside the context of SI have been observed in groups of recent suicide attempters [56]. The Cusp catastrophe model [57] describes how the transition from SI to suicidal behavior may occur through sudden shifts from low-risk to high-risk states, even in the absence of conscious SI but in the presence of a high capability for suicidal acts. In this study, when exploring predischarge differences, we were unable to distinguish between those who later did vs did not endorse momentary SI after discharge. A 10-day observation period provides only a snapshot of the circumstances patients face after discharge. Ultimately, participants should be followed over extensive periods in observational studies to determine who goes on to exhibit suicidal behavior.

#### Frequency of Peak Scores of Momentary SI

Armey et al [23] reported that participants hospitalized with SI were more likely to experience momentary SI compared to a group of controls. At the same time, participants hospitalized after an SA did not show different odds of experiencing SI in the postdischarge period compared with controls [23]. These findings are partly in line with the present results, where participants hospitalized with severe risk of suicide exhibited both a higher frequency of SI and a higher frequency of peak scores on SI. Our findings further suggested that momentary SI was characterized by a more state-dependent (reactive and context-dependent) pattern among those with a recent history of SA compared to those without. The theoretical ideation-to-action framework conceptualizes components that may explain why some people transition from thinking about suicide to acting on suicidal thoughts [58,59]. Erratic shifts in SI, driven by affective and situational or contextual factors in combination with a high capability for suicide, may represent a typical high-risk profile [16,23,58,59]. The 2 participant groups (those hospitalized following an SA and those hospitalized due to severe risk of suicide) may represent partly distinct and partly overlapping populations in terms of capability (attempt) and the mechanisms driving suicide risk and SI. Consistent with prior findings, we expected those with a recent history of SA to experience more frequent SI. These counterintuitive findings may be due to the small and heterogeneous sample available for analysis. Consequently, these observations should be replicated in future studies [23].

#### Baseline Variables That Predict the Intensity of Postdischarge Momentary SI

Two previous studies [23,30] have reported on the connection between SI before discharge and momentary SI after discharge. As noted earlier, Armey et al [23] observed a higher likelihood of SI after discharge among participants hospitalized with SI. Rogers et al [30] investigated how the frequency, perceived controllability, intensity, and duration of SI before discharge were associated with momentary SI after discharge. Participants’ perceived controllability of SI at baseline was associated with later momentary SI and SAs at a 3-week follow-up, whereas the frequency, intensity, and duration of baseline SI were not.

In the present study, we observed a significant association only between baseline levels on the self-assessed SSF-IV chronic subscale and momentary SI, but not between the clinician-administered BSSI-C and momentary SI. Interestingly, a few prior studies have reported low agreement between self-assessed and expert-assessed levels of SI [60,61], which may help explain this discrepancy. Evaluations of suicide risk guided by structured interviews may capture a phenomenon akin to “expert assessments of objective risk of suicide,” which may only partly correspond to patients’ lived experiences. A closer association between self-reported SI before and after discharge might be expected, as the EMA protocol samples lived experiences. The SSF-IV may be a valuable clinical assessment and treatment tool during hospitalization, as findings indicate a close relationship between its underlying components and postdischarge momentary SI. In this context, the SSF-IV may be used collaboratively by therapists and patients to inform understanding of the patient’s situation and to support treatment planning for the postdischarge period [62,63].

### Limitations

This study was exploratory and included only a small to moderate sample size. In addition, the sample size achieved was smaller than initially planned, resulting in reduced statistical power. In general, all results are tentative and hypothesis-generating, and the indicated associations should be subjected to further research in larger studies.

To identify baseline variables that predicted momentary SI, we explored associations from several complementary angles, which strengthened our findings. We refrained from running a final multiple regression analysis due to concerns about multicollinearity. Because of our small sample size, we did not examine the associations between individual PDs and momentary SI.

It is challenging to engage people from vulnerable groups in research, such as those patients at high risk of suicide [64]. Patients with high morbidity may not have been attainable to include in our study; they may have been too severely ill, prone to declining participation, or likely to drop out early, and clinicians may have been reluctant to refer them to study staff. A general question is whether this study, and other similar studies alike, succeeds in recruiting a representative and ecologically valid sample from the target group. A biased sample limits the broader generalizability of the findings. On the other hand, this project excluded participants with no planned follow-up in specialized mental health care services after discharge. This excluded subgroup was likely characterized by milder and less complex symptoms, as they were not in need of further specialized care. Their exclusion from the study may partly explain the higher occurrence of momentary SI observed in this study compared with prior research.

Missing surveys are common in momentary sampling studies [17,18]. Strengths of this study were the balanced gender distribution and the moderate response rate of 60% to the EMA surveys. In previous studies that followed patients during the first days after hospital discharge, compliance rates were generally low for most studies, varying between 20% and 34% [23,26-30], with the exception of a high completion rate of 74% in the French study [22]. Participants’ reasons for missing surveys may have been multifaceted, including motivation, technical issues, symptomatology, or other factors.

### Conclusions

This study offers a unique examination of how participants’ clinical status before discharge is related to short-term momentary SI after discharge. Discharge decisions are typically based on clinical judgment, while empirical knowledge on how patients are likely to react, feel, and cope remains limited. Clinicians should be aware that patients’ subjective distress may endure after hospitalization to a greater extent than previously reported in the literature, particularly among groups characterized by severe and complex symptoms and frequent prior SAs. Future research should further prioritize identifying patterns and dynamics of SI in high-risk populations and seek to distinguish profiles with different clinical needs. In this way, research may enable clinicians to intervene proactively rather than reactively and to tailor treatments and follow-up services in alignment with individuals’ needs. The SSF-IV may be a promising instrument to implement as a clinical assessment and treatment tool during hospitalization, which may guide both clinical judgments and treatment planning. Our observations regarding the potential of the SSF-IV chronic subscale to predict SI after discharge should be replicated in future research.

In light of our observations, individualized, timely, and easily accessible supportive follow-up services, psychoeducation, development of coping strategies, and safety planning are all well-founded treatment strategies during the first weeks after discharge from the hospital [65,66].

## Supplementary material

10.2196/88745 Multimedia Appendix 1 Workflow and interface of the mobile app.

10.2196/88745 Multimedia Appendix 2 Modeling of the time variable “day.”

10.2196/88745 Multimedia Appendix 3 Peak frequency of suicidal ideation and associations with baseline variables.

10.2196/88745 Multimedia Appendix 4 Baseline variables predictive associations with suicidal ideation adjusted for gender and age.

## Abbreviations

BSSI-C: Beck Scale for Suicide Ideation-Current
EMA: ecological momentary assessment
ICC: intraclass correlation coefficient
MAR: missing at random
MCAR: missing completely at random
OQ-45: Outcome Questionnaire-45
OR: odds ratio
PD: personality disorder
PHQ-9: Patient Health Questionnaire-9
SA: suicide attempt
SASIC: Suicide Attempt Self-Injury Count
SCID-5-PD: Structured Clinical Interview for *DSM-5* Personality Disorders
SI: suicidal ideation
SSF-IV: Suicide Status Form-IV
