# Supplementary material for: Suicidal Ideation After Discharge From Psychiatric Hospital: Momentary Assessment Study
Source: JMIR Ment Health. 2026 Jul 31;13:e88745. doi: 10.2196/88745 (PMC13427071; doi:10.2196/88745)

## Slide 1
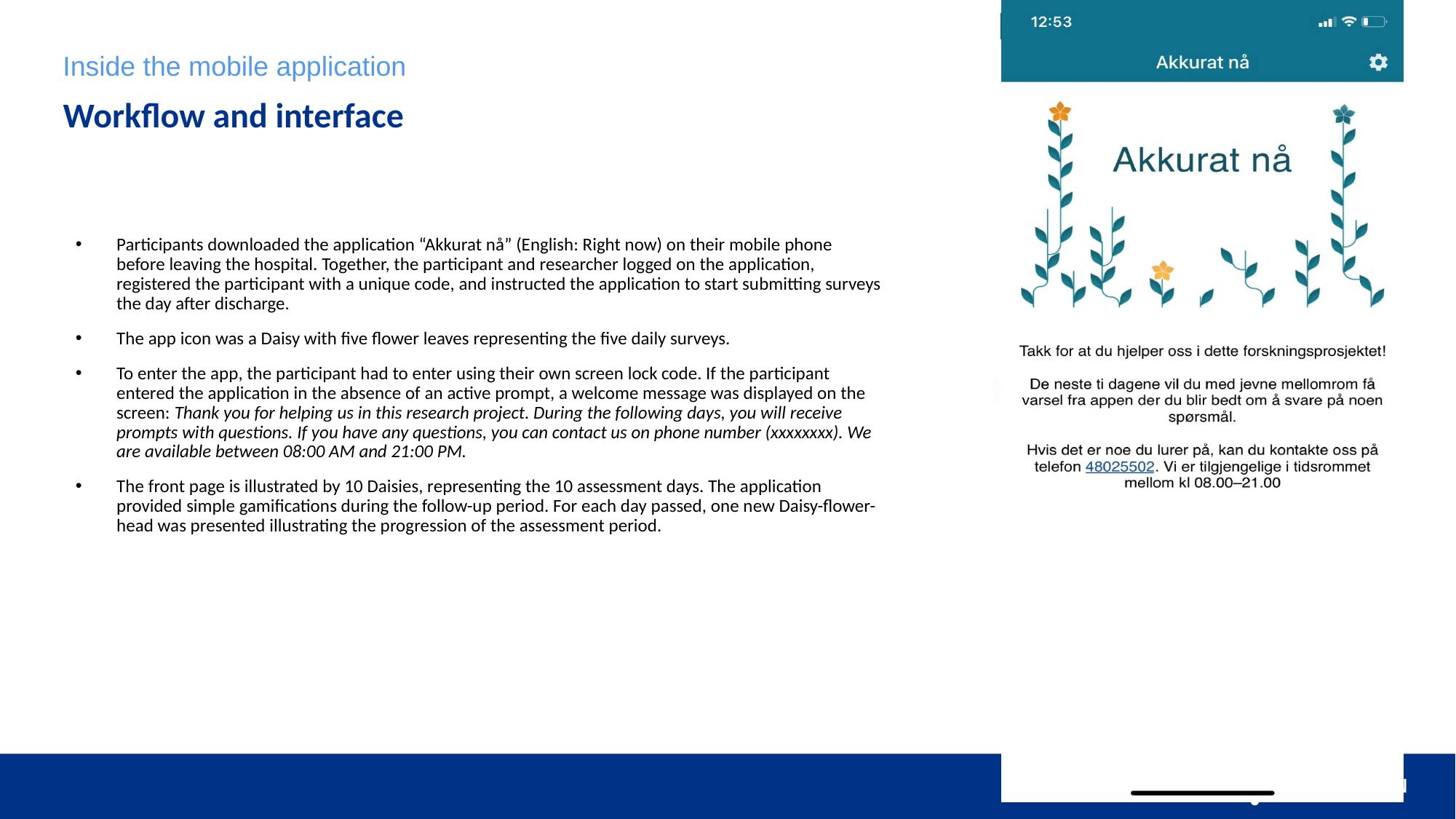

Inside the mobile application
# Workflow and interface
Participants downloaded the application “Akkurat nå” (English: Right now) on their mobile phone before leaving the hospital. Together, the participant and researcher logged on the application, registered the participant with a unique code, and instructed the application to start submitting surveys the day after discharge.
The app icon was a Daisy with five flower leaves representing the five daily surveys.
To enter the app, the participant had to enter using their own screen lock code. If the participant entered the application in the absence of an active prompt, a welcome message was displayed on the screen: Thank you for helping us in this research project. During the following days, you will receive prompts with questions. If you have any questions, you can contact us on phone number (xxxxxxxx). We are available between 08:00 AM and 21:00 PM.
The front page is illustrated by 10 Daisies, representing the 10 assessment days. The application provided simple gamifications during the follow-up period. For each day passed, one new Daisy-flower-head was presented illustrating the progression of the assessment period.

## Slide 2
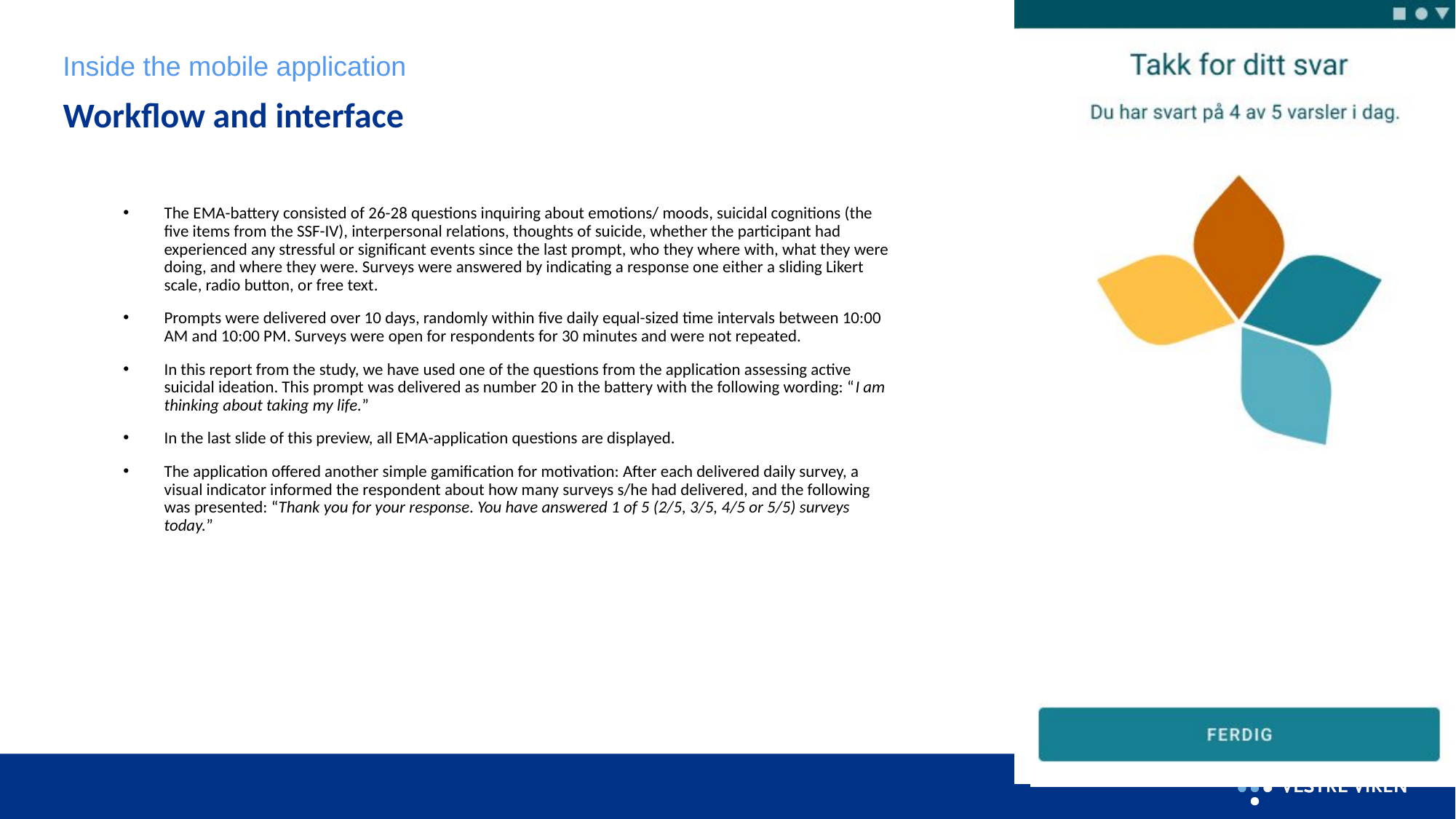

Inside the mobile application
# Workflow and interface
The EMA-battery consisted of 26-28 questions inquiring about emotions/ moods, suicidal cognitions (the five items from the SSF-IV), interpersonal relations, thoughts of suicide, whether the participant had experienced any stressful or significant events since the last prompt, who they where with, what they were doing, and where they were. Surveys were answered by indicating a response one either a sliding Likert scale, radio button, or free text.
Prompts were delivered over 10 days, randomly within five daily equal-sized time intervals between 10:00 AM and 10:00 PM. Surveys were open for respondents for 30 minutes and were not repeated.
In this report from the study, we have used one of the questions from the application assessing active suicidal ideation. This prompt was delivered as number 20 in the battery with the following wording: “I am thinking about taking my life.”
In the last slide of this preview, all EMA-application questions are displayed.
The application offered another simple gamification for motivation: After each delivered daily survey, a visual indicator informed the respondent about how many surveys s/he had delivered, and the following was presented: “Thank you for your response. You have answered 1 of 5 (2/5, 3/5, 4/5 or 5/5) surveys today.”

## Slide 3
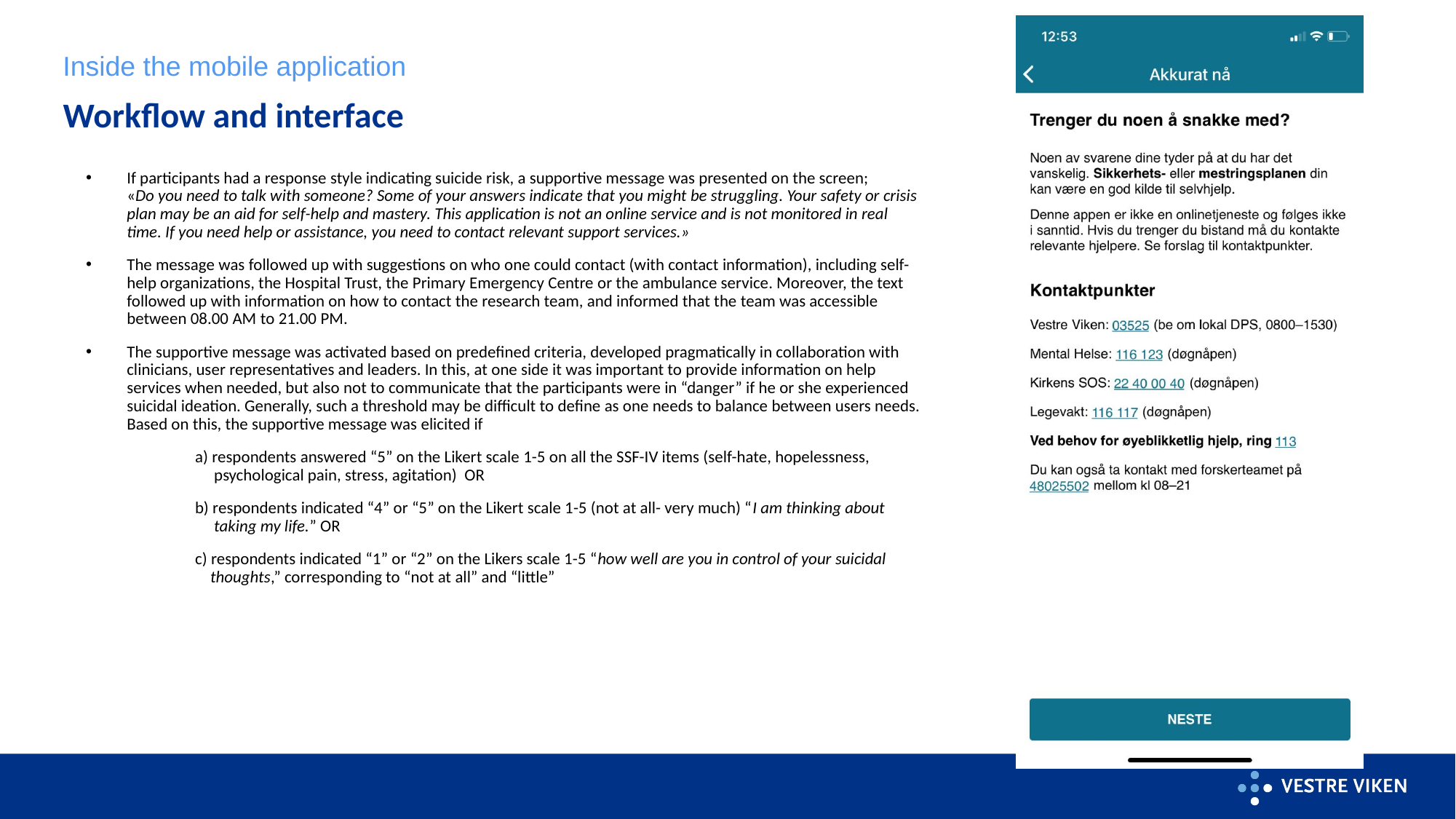

Inside the mobile application
# Workflow and interface
If participants had a response style indicating suicide risk, a supportive message was presented on the screen; «Do you need to talk with someone? Some of your answers indicate that you might be struggling. Your safety or crisis plan may be an aid for self-help and mastery. This application is not an online service and is not monitored in real time. If you need help or assistance, you need to contact relevant support services.»
The message was followed up with suggestions on who one could contact (with contact information), including self-help organizations, the Hospital Trust, the Primary Emergency Centre or the ambulance service. Moreover, the text followed up with information on how to contact the research team, and informed that the team was accessible between 08.00 AM to 21.00 PM.
The supportive message was activated based on predefined criteria, developed pragmatically in collaboration with clinicians, user representatives and leaders. In this, at one side it was important to provide information on help services when needed, but also not to communicate that the participants were in “danger” if he or she experienced suicidal ideation. Generally, such a threshold may be difficult to define as one needs to balance between users needs. Based on this, the supportive message was elicited if
	a) respondents answered “5” on the Likert scale 1-5 on all the SSF-IV items (self-hate, hopelessness, 	 psychological pain, stress, agitation) OR
	b) respondents indicated “4” or “5” on the Likert scale 1-5 (not at all- very much) “I am thinking about 	 taking my life.” OR
	c) respondents indicated “1” or “2” on the Likers scale 1-5 “how well are you in control of your suicidal 	 thoughts,” corresponding to “not at all” and “little”

## Slide 4
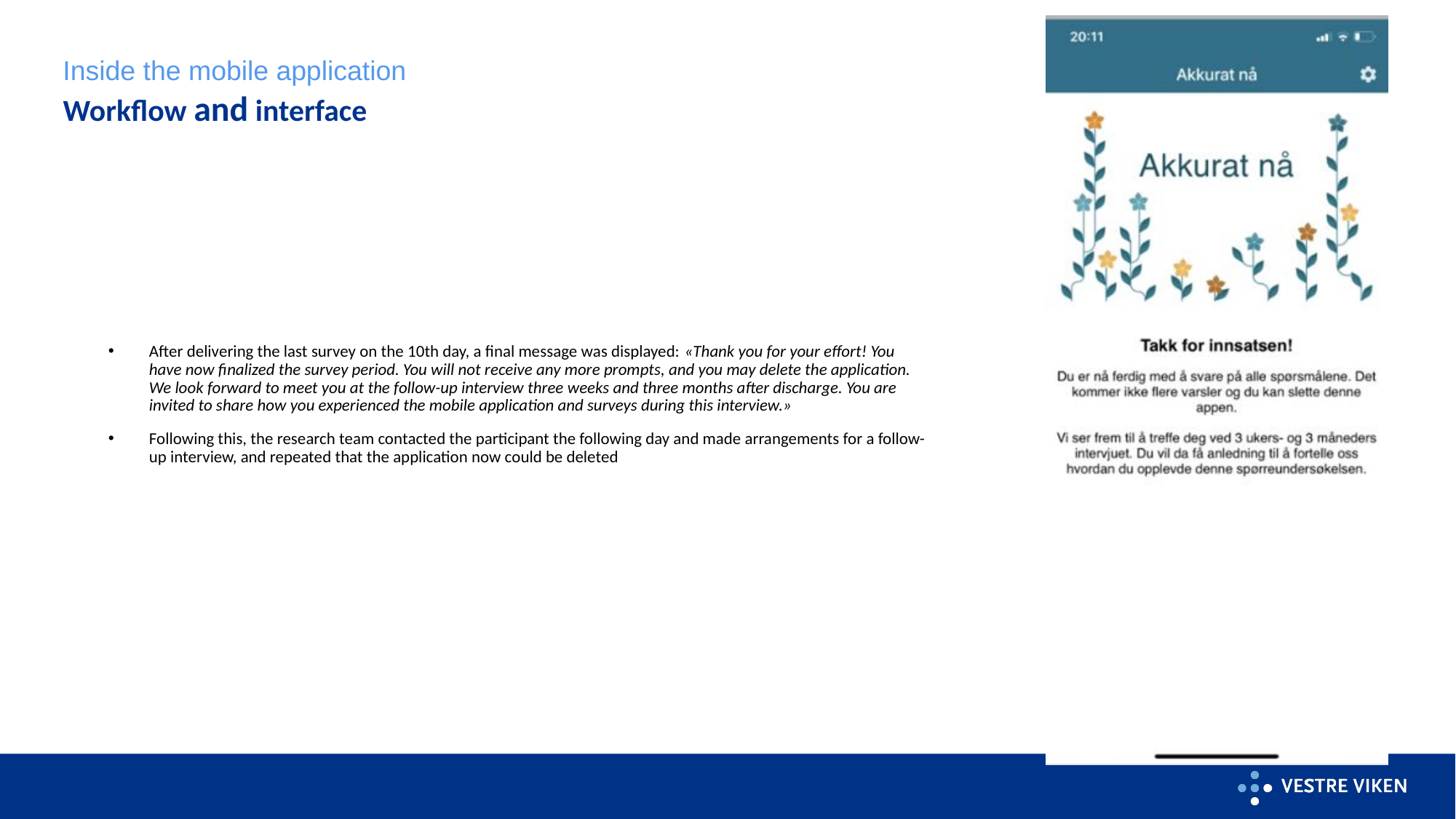

Inside the mobile application
# Workflow and interface
After delivering the last survey on the 10th day, a final message was displayed: «Thank you for your effort! You have now finalized the survey period. You will not receive any more prompts, and you may delete the application. We look forward to meet you at the follow-up interview three weeks and three months after discharge. You are invited to share how you experienced the mobile application and surveys during this interview.»
Following this, the research team contacted the participant the following day and made arrangements for a follow-up interview, and repeated that the application now could be deleted

## Slide 5
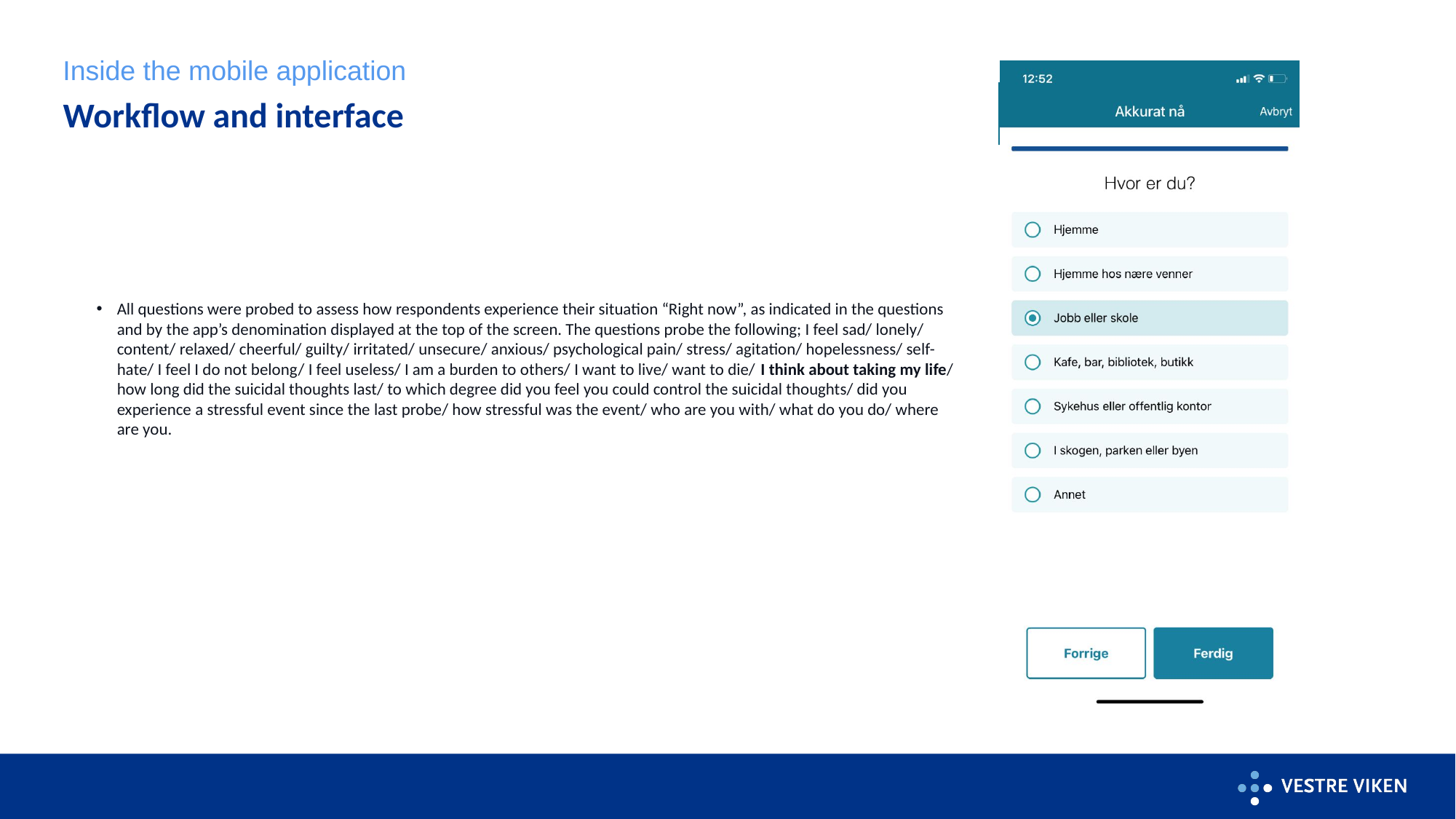

Inside the mobile application
# Workflow and interface
All questions were probed to assess how respondents experience their situation “Right now”, as indicated in the questions and by the app’s denomination displayed at the top of the screen. The questions probe the following; I feel sad/ lonely/ content/ relaxed/ cheerful/ guilty/ irritated/ unsecure/ anxious/ psychological pain/ stress/ agitation/ hopelessness/ self-hate/ I feel I do not belong/ I feel useless/ I am a burden to others/ I want to live/ want to die/ I think about taking my life/ how long did the suicidal thoughts last/ to which degree did you feel you could control the suicidal thoughts/ did you experience a stressful event since the last probe/ how stressful was the event/ who are you with/ what do you do/ where are you.

## Slide 6
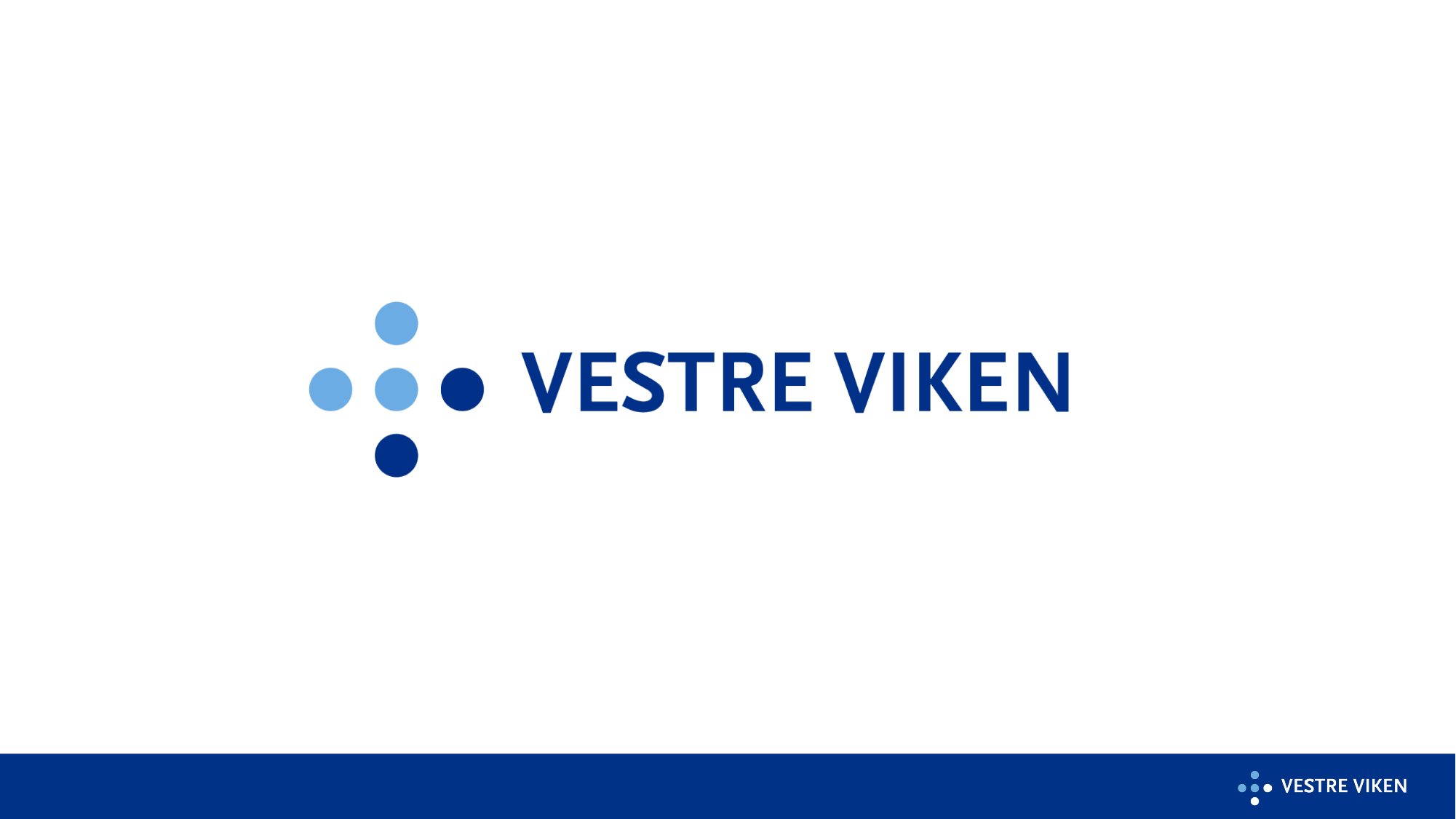

Supplement: Multimedia Appendix 1 [file mental-v13-e88745-s001.pptx]
