# Supplementary material for: Suicidal Ideation After Discharge From Psychiatric Hospital: Momentary Assessment Study
Source: JMIR Ment Health. 2026 Jul 31;13:e88745. doi: 10.2196/88745 (PMC13427071; doi:10.2196/88745)
Supplement: Multimedia Appendix 2 [file mental-v13-e88745-s002.docx]

**Supplementary Appendix.** Modelling of the time variable “day”

We first modelled whether SI changed significantly from day to day, in a categorical model. Overall, we observed no significant effects. Thereafter, we fitted a spline model and observed that changes in slopes were not significant. Thereafter, we modelled time as continuous and again did not observe a significant change in SI. We explored also whether a categorical model was a better fit than a linear model but could not observe a significant difference. See Figure and Table below, that illustrates the substantial overlapping slopes of SI when adopting categorical, linear or spline model.

Overview of time models

| **Specification of day** | **Purpose/ assumptions** | **Pros and cons** | **Result** |
| --- | --- | --- | --- |
| Categorical (categorical) | Allows unrestricted day-specific means | Require adequate sample size to calculate the regression coefficient for each day compared to the reference day | No stat. sign. effect of days as categoric |
| Spline (4 segments; 3 knots) | Allows non-linear change over time | Require a lot of data to calculate the slopes for the segments in the fixed part and the random part. | The estimates of the slopes were not stat. sign |
| Linear (continuous) | Assumes a constant rate of change across days | Require only to estimate one regression coefficient (i.e. the slope) for day. | No stat. sign. effect of days as continuous |

Time models and comparisons with test statistics

|  | Effect of time | df | χ² | *P*-value | AIC | BIC |
| --- | --- | --- | --- | --- | --- | --- |
|  |  |  |  |  |  |  |
| Days as piecewise linear spline, different random slope for different segment | No significant slope | 21 |  |  | 2224.4 | 2329.0 |
|  |  |  |  |  |  |  |
| Days as categorical | no significant effect of days | 14 | χ²(8) = 10.9 | 0.206 | 2257.0 | 2326.7 |
| Days as continuous | no significant effect of days | 6 |  |  | 2251.9 | 2281.8 |

Illustration of SI trajectories depicting categorical, spline and linear time model

“Day” modelled as either linear/ continuous, categorical or in a spline model specified with four-time segments with three knots. All models substantially overlapped, indicating no meaningful significance of non-linearity. Blue color: day modelled as continuous/ linear, Black color: day as 4 segments for 3 knots, Red color: day modelled as categorical

**Conclusion:** The mixed model with categorical, continuous and piecewise linear splines as fixed variables showed no significant effect of time. The piecewise linear splines model seems to be the best compared with the other models since the AIC value is lowest, but the model required a lot of data to estimate the fixed and random coefficients. Thus, BIC values seem to favor the continuous model. There was also no statistically significant difference between the model with days as categorical and the model with days as continuous ( χ²(8) = 10.9 , *P*= 0.206). In conclusion, for parsimony, we chose to carry on with the linear model in further analyses.
