# Supplementary material for: Suicidal Ideation After Discharge From Psychiatric Hospital: Momentary Assessment Study
Source: JMIR Ment Health. 2026 Jul 31;13:e88745. doi: 10.2196/88745 (PMC13427071; doi:10.2196/88745)
Supplement: Multimedia Appendix 3 [file mental-v13-e88745-s003.docx]

|  | **Unadjusted** | | **Adjusted** | |
| --- | --- | --- | --- | --- |
| **Variables** | **Beta (95% CI)** | ***P-*value^a^** | **Beta (95% CI)** | ***P-*value^a^** |
| Males | −5.00 (−15.76 to 5.78) | .35 | −4.08 (−16.13 to 7.96) | .50 |
| Age | −.05 (−.57 to .47) | .85 | .08 (−.41 to .57) | .73 |
| Children, yes | 1.00 (−11.33 to 13.33) | .87 | .42 (−15.03 to 15.86) | .96 |
| Previous hospitalizations | .31 (−.03 to .64) | .07 | .27 (−.10 to .64) | .14 |
| Days of hospitalization | .10 (−.33 to .53) | .64 | .04 (−.38 to .45) | .55 |
| Diagnoses of depression, yes | 2.00 (−.9.78 to 13.78) | .73 | −.42 (−11.62 to 10.78) | .94 |
| Personality disorder, yes | 4.00 (−5.78 to 13.78) | .41 | 1.30 (−11.02 to 13.61) | .83 |
| Suicide attempt (SA) lifetime | .20 (−.14 to .55) | .24 | .17 (−17 to .52) | .31 |
| SA prior to hospitalization, yes | 16.00 (8.05 to 23.95) | .001 | 17.74 (8.56 to 26.91) | .001 |
| Severe habitual self-harm^b^, yes | −12.00 (−24.88 to .88) | .067 | −12.89 (27.53 to 1.75) | .08 |
| Suicide ideation (BSSI-C ^c^), mean (SD) | .36 (−.86 to 1.59) | .55 | .06 (−1.14 to 1.27) | .92 |
| Symptom severity (OQ-45^d^), mean (SD) | .04 (−.23 to .31) | .74 | .07 (−.17 to .31) | .56 |
| Depression (PHQ-9 ^e^), mean (SD) | .31 (−.73 to 1.34) | .55 | .20 (−.82 to 1.22) | .69 |
| Suicidal cognitions (SSF-IV ^f^), mean (SD) | .46 (−.59 to 1.51) | .38 | .30 (−.74 to 1.34) | .57 |
| SSF-IV acute-subscale, mean (SD) | .00 (−3.00 to 3.00) | 1.00 | .20 (−.2.30 to 2.71) | .87 |
| SSF-IV chronic-subscale, mean (SD) | 1.00 (−.35 to 2.35) | .14 | .44 (−1.07 to 1.96) | .55 |
| SSF-IV self-assessed suicide risk, mean (SD) | 7.00 (3.52 to 10.48) | .001 | 6.95 (3.95 to 9.95) | .001 |

**Table S1** Peak Frequency of Suicidal ideation and associations with baseline variables

^a^ Bonferroni correction for multiple teste: .005/ 17= .00294 ^b^Subpopulation who were unable to quantify self-harm episodes (< 100), ^c^BSSI-C= Beck Scale for Suicide Ideation – Current, ^d^OQ-45= Outcome Questionnaire-45, ^e^PHQ-9= Patient Health Questionnaire-9, ^f^SSF-IV= Suicide Status Form-IV
