# Supplementary material for: Suicidal Ideation After Discharge From Psychiatric Hospital: Momentary Assessment Study
Source: JMIR Ment Health. 2026 Jul 31;13:e88745. doi: 10.2196/88745 (PMC13427071; doi:10.2196/88745)
Supplement: Multimedia Appendix 4 [file mental-v13-e88745-s004.docx]

**Table S1.** Baseline variables potential predictive associations with momentary suicide ideation. Results from a series of mixed regression analyses

|  |  | **Unadjusted** | | **Adjusted for gender and age** | |
| --- | --- | --- | --- | --- | --- |
| **Baseline variables** | **Parameter** | **Beta^a^ (95% CI)** | ***P^g^*- value** | **Beta^a^ (95% CI)** | ***P^g^*- value** |
| Model 1) Gender | Day | .05 (.00 to .10) | .04 | .05 (.00 to .10) | .04 |
|  | Males | .47 (−.15 to 1.09) | .13 | .41 (−.27 to 1.08) | .23 |
|  | Day* males | .05 (−.11 to .02) | .20 | −.05 (−.11 to .02) | .19 |
| Model 2) Age | Day | −.05 (−.16 to .07) | .43 | −.05 (−.16 to .07) | .43 |
|  | Age | −.02 (−.04 to .01) | .24 | −.01 (−.04 to .02) | .48 |
|  | Day* age | .00 (−.00 to .00) | .17 | .00 (−.00 to .00) | .17 |
| Model 3) Children | Day | −.00 (−.05 to .05) | .92 | −.00 (−.05 to .05) | .93 |
|  | Children (yes) | −.47) (−.1.08 to .16) | .14 | −.29) (−.1.16 to .57) | .49 |
|  | Day* children | .06 (.00 to .13) | .07 | .06 (−.00 to .13) | .07 |
| Model 4) Hospitalizations, count | Day | .02 (−.01 to .06) | .20 | .02 (−.01 to .06) | .20 |
|  | Hospitalizations | .02 (−.00 to .00) | .11 | .02 (−.00 to .04) | .12 |
|  | Day* hospitalizations | .00 (−.00 to .00) | .50 | .00 (−.00 to .00) | .50 |
| Model 5) Days of hospitalization | Day | −.01 (−.06 to .04) | .78 | −.01 (−.06 to .04) | .78 |
|  | Days of hospitalization | .01 (.03 to .01) | .46 | .01 (−.03 to .02) | .46 |
|  | Day* days of hospitalization | .00 (.00 to .01) | .07 | .00 (.00 to .01) | .07 |
| Model 6) Diagnoses of depression | Day | .04 (−.01 to .09) | .15 | .04 (−.01 to .09) | .15 |
|  | Diagnoses of depression | .43 (−1.06 to .19) | .17 | −.37 (−1.00 to .27) | .25 |
|  | Day* diagnoses of depression | −.01(−.08 to .06) | .71 | −.01(−.08 to .06) | .72 |
| Model 7) Personality disorder | Day | .07 (.00 to .13) | .04 | .07 (.00 to .13) | .04 |
|  | Personality disorder | .27 (−.48 to 1.02) | .47 | .20 (−.48 to 1.02) | .61 |
|  | Day* personality disorder | −.06 (−.14 to .03) | .18 | −.06 (−.14 to .03) | .18 |
| Model 8) Reason for hospitalization (SA^b^) | Day | .03 (−.03 to .09) | .26 | .03 (−.03 to .09) | .26 |
|  | Reason for hospitalization | .49 (−1.15 to .17) | .14 | .50 (−1.15 to .16) | .13 |
|  | Day* reason for hospitalization | −.01(−.08 to .07) | .87 | −.01(−.08 to .07) | .88 |
| Model 9) Suicide attempt lifetime | Day | .03 (−.00 to .07) | .07 | .03 (−.00 to .07) | .07 |
|  | Suicide attempt lifetime | .02 (.01 to .04) | .008 | .02 (.01 to .04) | .009 |
|  | Day* suicide attempt lifetime | −.00 .40 (−.00 to .00) | .40 | −.00 (−.00 to .00) | .40 |
| Model 10) Severe habitual self- harm (HSH) | Day | .04 (−.01 to .08) | .08 | .04 (−.01 to .08) | .08 |
|  | HSH | .41 (−.35 to 1.17) | .28 | .23(−.35 to 1.17) | .59 |
|  | Day* HSH | −.02 (−.11 to .06) | .56 | −.03 (−.64 to 1.11) | .56 |
| Model 11) Suicide ideation (BSSI-C^c^) | Day | .04 (−.15 to 23) | .68 | .04 (−.15 to .23) | .67 |
|  | BSSI-C | .04 (−.03 to .11) | .22 | .04 (−.03 to .11) | .23 |
|  | Day* BSSI-C | .00 (−.01 to .01) | .92 | .00 (−.01 to .01) | .91 |
| Model 12) Symptom severity (Oq-45^d^) | Day | −.04 (−.19 to .12) | .64 | −.04 (−.19 to .12) | .64 |
|  | OQ-45 | .02 (.01 to .03) | .006 | .02 (.01 to .03) | .009 |
|  | Day* OQ-45 | .00 (−.00 to .00) | .39 | .00 (−.00 to .00) | .39 |
| Model 13) Depression (PHQ-9^e^) | Day | −.02 (−.17 to .13) | .80 | −.02 (−.16 to .13) | .81 |
|  | PHQ-9 | .05 (−.01 to .11) | .11 | .06 (−.01 to .12) | .08 |
|  | Day* PHQ-9 | .00 (−.00 to .01) | .50 | .00 (−.01 to . 12) | .50 |
| Model 14) Suicide Status Form (SSF-IV^f^) | Day | −.07 (−.19 to .04) | .20 | −.07 (−.19 to .04) | .20 |
|  | SSF-IV | .09 (.03 to .14) | .004 | .09 (.03 to .15) | .004 |
|  | Day* SSF-IV | .01 (.00 to .01) | .07 | .01 (.00 to .01) | .07 |
| Model 15) SSF-IV subscale "chronic" | Day | −.05(−.15 to .05) | .33 | −.05 (−.15 to .05) | .33 |
|  | SSF-IV chronic | .13 (.05 to .20) | .003 | .13 (.05 to .21) | .002 |
|  | Day* SSF-IV chronic | .01 (−.00 to .02) | .11 | .01 (−.00 to .02) | .11 |
| Model 16) SSF-IV "acute" | Day | −.06 (−.16 to .05) | .29 | −.06 (−.16 to .05) | .29 |
|  | SSF-IV acute | .13 (−.01 to .26) | .07 | .12 (−.02 to .27) | .10 |
|  | Day* SSF-IV acute | .01 (−.00 to .03) | .09 | .01 (−.00 to .03) | .09 |
| Model 17) SSF-IV self-assessed suicide risk | Day | −.02 (−.10 to .06) | .61 | −.01 (−.09 to .06) | .72 |
|  | Suicide risk | .20 (−.02 to .41) | .07 | .18 (−.04 to .40) | .10 |
|  | Day*suicide risk | .02 (.01 to .05) | .17 | .02 (−.01 to .05) | .21 |

^a^Beta coefficient from mixed regression analyses ^b^SA= Suicide attempt, ^c^BSSI-C= Beck Scale for Suicide Ideation – Current, ^d^OQ-45= Outcome Questionnaire-45, ^e^PHQ-9= Patient Health Questionnaire-9, ^f^SSF-IV= Suicide Status Form-IV.

^g^Bonferroni correction for multiple tests *P*= .05/ 17 = .00294
